# Automatic Speech Discrimination Assessment Methods Based on Event-Related Potentials (ERP)

**DOI:** 10.3390/s22072702

**Published:** 2022-04-01

**Authors:** Pimwipa Charuthamrong, Pasin Israsena, Solaphat Hemrungrojn, Setha Pan-ngum

**Affiliations:** 1 Interdisciplinary Program of Biomedical Engineering, Faculty of Engineering, Chulalongkorn University, Pathumwan, Bangkok 10330, Thailand; pimwipa.c@alumni.chula.ac.th; 2 National Electronics and Computer Technology Center, 112 Thailand Science Park, Klong Luang, Pathumthani 12120, Thailand; pasin.israsena@nectec.or.th; 3 Department of Psychiatry, Faculty of Medicine, Chulalongkorn University, Pathumwan, Bangkok 10330, Thailand; solaphat.h@chula.ac.th; 4 Department of Computer Engineering, Faculty of Engineering, Chulalongkorn University, Pathumwan, Bangkok 10330, Thailand

**Keywords:** EEG, ERP, speech discrimination, classifier

## Abstract

Speech discrimination is used by audiologists in diagnosing and determining treatment for hearing loss patients. Usually, assessing speech discrimination requires subjective responses. Using electroencephalography (EEG), a method that is based on event-related potentials (ERPs), could provide objective speech discrimination. In this work we proposed a visual-ERP-based method to assess speech discrimination using pictures that represent word meaning. The proposed method was implemented with three strategies, each with different number of pictures and test sequences. Machine learning was adopted to classify between the task conditions based on features that were extracted from EEG signals. The results from the proposed method were compared to that of a similar visual-ERP-based method using letters and a method that is based on the auditory mismatch negativity (MMN) component. The P3 component and the late positive potential (LPP) component were observed in the two visual-ERP-based methods while MMN was observed during the MMN-based method. A total of two out of three strategies of the proposed method, along with the MMN-based method, achieved approximately 80% average classification accuracy by a combination of support vector machine (SVM) and common spatial pattern (CSP). Potentially, these methods could serve as a pre-screening tool to make speech discrimination assessment more accessible, particularly in areas with a shortage of audiologists.

## 1. Introduction

Pure-tone audiometry (PTA) and speech audiometry are routinely used in a clinical setting to assess auditory function [1]. PTA measures the minimum threshold level that can be heard by the user at different frequencies. As PTA only evaluates the absolute hearing threshold but not the ability to recognize speech, speech audiometry is used as a complement to PTA in order to measure different aspects of a patient’s auditory function altogether. Speech audiometry commonly includes three speech tests: speech-detection threshold (SDT), speech reception threshold (SRT), and speech discrimination. SDT measures the threshold at which a patient can detect the presence of speech 50% of the time. SRT represents the threshold at which a patient can repeat 50% of the speech. Both SDT and SRT can be determined in a similar way to PTA but use speech instead of pure-tone sounds. Speech discrimination is more complex to determine. A commonly used method to assess speech discrimination includes presenting monosyllabic words at 50 dB above SRT and measure the percentage of correctly repeated words [2]. Speech discrimination scores, along with results from other tests, are used in diagnosing and determining treatment for hearing loss patients [2]. However, the behavioral assessment to test speech discrimination requires subjective responses which makes the assessment more difficult in some cases such as difficult-to-test patients or children. An electrophysiological method to assess speech discrimination provides an objective assessment and would be suitable in these situations.

Electroencephalography (EEG) measures the electrical potentials at the scalp which reflect the brain’s electrical activity [3]. An EEG test usually includes placing electrodes on the scalp. The placement of the electrodes usually follows the 10–20 system [4] which indicates the different positions of the scalp using a combination of letters and numbers. For higher resolution, the ten percent electrode system [5] or the five percent electrode system [6] which can accommodate a larger number of electrodes could also be used. EEG signals have high temporal resolution compared to other modalities (e.g., functional magnetic resonance imaging or functional near infrared spectroscopy) and directly reflects neural activity [7]. For these reasons, EEG is a well-suited technique to study cognition as it can capture the rapid dynamics of cognitive processes which happen in the order of milliseconds. To analyze EEG signals, raw signals are often grouped into bands including delta (2–4 Hz), theta (4–8 Hz), (8–12 Hz), beta (15–30 Hz), and gamma (30–150 Hz) [7]. EEG changes that are triggered by specific events or stimuli are termed event-related potentials (ERPs). ERPs can be related to visual, auditory, or somatosensory stimuli [8]. ERP waveforms are obtained from averaging across many trials to extract the response that is related to the stimuli. The observed ERP waveforms that are recorded from the scalp usually represent the sum of multiple ERP components [9].

A commonly studied ERP component that is related to speech discrimination is the mismatch negativity (MMN) [10,11,12]. MMN is an ERP component that is elicited by a deviant stimulus that violates the representation of the standard stimulus that is formed by repetition [11]. MMN is conventionally studied using an oddball paradigm which usually involves a sequence of repeated standard stimuli, which occurs in most of the trials, sporadically interrupted by a deviant stimulus. This component has been used in various work that is related to auditory discrimination and speech discrimination [13,14,15,16,17,18]. The presence of MMN in response to the deviant stimuli in an oddball task indicates that the user can distinguish between the particular deviant and standard sounds that are used in the task. As MMN can be elicited even when users are not paying attention to the sound, an MMN-based method to assess speech discrimination is well-suited for a variety of participants, particularly those who have difficulties following instructions. However, an oddball task that is used to elicit MMN typically uses only one pair of sounds. Word lists that are used to test speech discrimination in Thai usually consist of 20–50 words [19,20,21]. For example, the word list that was proposed by Yimtae et al. [21] contained 24 words, the word list that was used by Visessumon [20] had 21 words, and the word list that was proposed by Hemakom et al. [19] comprised of 45 words. To test speech discrimination using an MMN-based method, the participants would be required to undergo many rounds of oddball tasks with different standard and deviant stimuli which would be time-consuming and repetitive. These limitations have led to various improvements and alternatives being proposed such as variations to the oddball paradigm [15,22], different analysis approaches [23,24], or an alternative method or marker [25,26].

Morikawa et al. [26] proposed an alternative speech discrimination assessment method that utilized visual ERP that was induced by visual stimuli that was associated with an auditory stimuli instead of auditory ERP. In their method, the participants listened to a sound corresponding to a Japanese letter and then were shown a picture of a Japanese letter. In 50% of the trials, the picture matched the letter that was presented. In the remaining trials, the picture matched another letter that had similar pronunciation and was often confused with the former letter. The participants were required to answer whether the picture and the sound matched. The study only included participants with normal hearing but some of the auditory stimuli that were used were manipulated to imitate hearing loss. They found that when the participants answered that the sound and picture matched (match condition), an ERP component called the P3 [27,28] was elicited at 290–400 ms after the visual stimulus onset. P3 was usually observed during stimulus discrimination and was theorized to be related to the brain activities that updated a mental model when a new stimulus was detected. When the participants answered that the sound and picture did not match (mismatch condition), a late positive potential (LPP) [29] was elicited at 480–570 ms after visual stimulus onset. LPP was assumed to be similar to the P3b component [28,30] and was elicited when there was a mismatch between the expectation and feedback. To classify between the match and mismatch conditions, Morikawa et al. [26] calculated a feature value from the average amplitude difference between the intervals where P3 and LPP were observed. This feature value was compared to a predetermined threshold value to separate between cases with successful and unsuccessful discrimination. They reported achieving 70.5% accuracy when one trial was used and more than 80% accuracy when four or more trials were averaged together. The promising result suggested that the proposed method might be a viable alternative to an MMN-based method. However, this method was only applicable to participants who can identify the Japanese letters. Therefore, the method might not be accessible to young children or illiterate people.

Currently, there is no widely used method to automatically assess speech discrimination. An ERP-based method provides an objective assessment of speech discrimination and could make the test more accessible, particularly in areas where there is a shortage of audiologists. An MMN-based method that is employed in many studies would be well-suited for assessing speech discrimination in children or patients who have difficulties with the behavioral test. However, as mentioned, this method might be time-consuming as many rounds of oddball task are needed in order to get an assessment that covers all the meaningful contrasts in a language. A possible alternative to an MMN-based method is a visual-ERP-based method that was proposed by Morikawa et al. [26] which was reported to have high accuracy. However, the use of Japanese letters limited the use of this method to literate patients only. Furthermore, there had not been an accuracy level obtained from an MMN-based method for comparison. Our research proposed a modification to the visual-ERP-based method by using pictures representing word meaning to make the method more accessible. This modified method was compared to the original visual-ERP-based method and an MMN-based method. In each method, machine learning techniques were employed to separate between the two different conditions. We hoped to recommend a suitable method that is based on ERP components, discrimination accuracy, and time efficiency. An overall framework combining a suitable ERP method and a classification technique into an automatic speech discrimination assessment system was also proposed.

## 2. Materials and Methods

### 2.1. Participants

A total of 30 native Thai volunteers participated in the research (mean age = 31 years, age range = 19–43 years, 13 males, 17 females). All of them had self-reported normal hearing and normal or corrected-to-normal vision. All the participants provided informed consent before beginning the research. The Research Ethics Review Committee for Research Involving Human at Chulalongkorn University approved the research protocol (protocol no. 171.1/63).

### 2.2. Procedure and Stimuli

There were two Thai words that were used in the experiment -/kài/(meaning chicken) and /k^h^ài/(meaning egg). Both words are basic nouns that are used in everyday life. The two words have the same vowel and tone but have different consonants. As Thai is a tonal language, tones are essential in distinguishing between words [31]. In this work, we aimed to test the ability to differentiate between words with consonant contrasts. Thus, the vowel and tone were the same for both words. The sounds were recorded by a native Thai woman and were obtained from The Thai Alphabets multimedia exhibit [32]. Each word had a duration of 500 ms with average sound pressure levels of approximately 60 dBSPL and 40 dBSPL as measured using a sound level meter. The stimuli were presented using PsychoPy [33].

The experiment consisted of three methods. From here on, the modified visual-ERP-based method that uses pictures of word meaning will be referred to as Method 1. The original visual-ERP-based method that was proposed by Morikawa et al. (2012) will be referred to as Method 2. The MMN-based method that only utilizes auditory stimuli will be referred to as Method 3.

The results from a pilot experiment suggested that using multiple pictures led to higher accuracy. However, using multiple pictures would also increase the experiment time considerably. Thus, we decided to further separate the picture method (Method 1) into three strategies. We aimed to compare between these strategies to find the optimal strategy in terms of both accuracy and time-efficiency. The three strategies included single-picture, multiple-pictures, and single-picture-with-expectation. We will call these Methods 1a, 1b, and 1c, respectively. The participants were randomly separated into three groups, with ten participants per group. Each group undertook either Method 1a, 1b, or 1c along with Method 2 and 3. The presentation order of the methods was counterbalanced across the participants. A laptop monitor and speaker were used to present the visual and auditory stimuli. The participants were seated approximately 0.3 m from the laptop and instructed to sit still to minimize movement artifacts.

**Figure 1:**
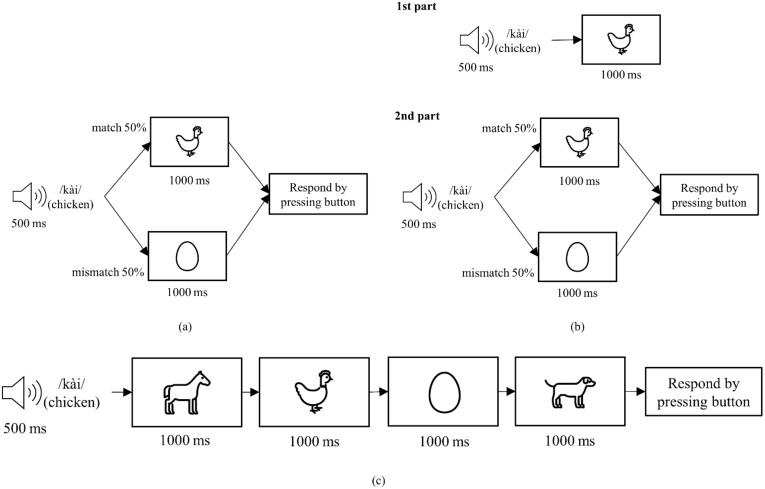
Event sequence for each trial in each strategy in Method 1 including (**a**) Method 1a; (**b**) Method 1c; (**c**) Method 1b.

Figure 1 shows the sequence of each trial in Method 1a, 1b, and 1c. For Method 1a, each trial started by playing a word through the laptop speaker for 500 ms. Then, a picture was shown for 1000 ms. There were 80 trials in this method, with the picture matching the meaning of the word in 40 trials. In the remaining trials, the picture did not match the meaning of the word. The participants were required to press ‘1’ on a standard keyboard if they think the picture matched the word they heard and press ‘2’ otherwise.

In Method 1b, the participants listened to a 500 ms long word then were shown a sequence of four pictures. The pictures were shown one-by-one for 1000 ms each. In each trial, one picture matched the meaning of the word that was played. The order of the correct picture was randomized, appearing as the first (or second, third, or fourth) picture in one fourth of the trials (12 trials out of 48 total trials). After seeing all four pictures, the participants were required to press ‘1’, ‘2’, ‘3’, or ‘4’ on the keyboard according to the position of the correct picture.

Method 1c consisted of two parts. In the first part, a 500 ms long word was played before a picture corresponding to the meaning of the word was shown for 1000 ms. This sequence was repeated 10 times for each word in a random order. This first part was added to show the participants the correct picture to expect for each word. The second part was identical to Method 1a.

**Figure 2:**
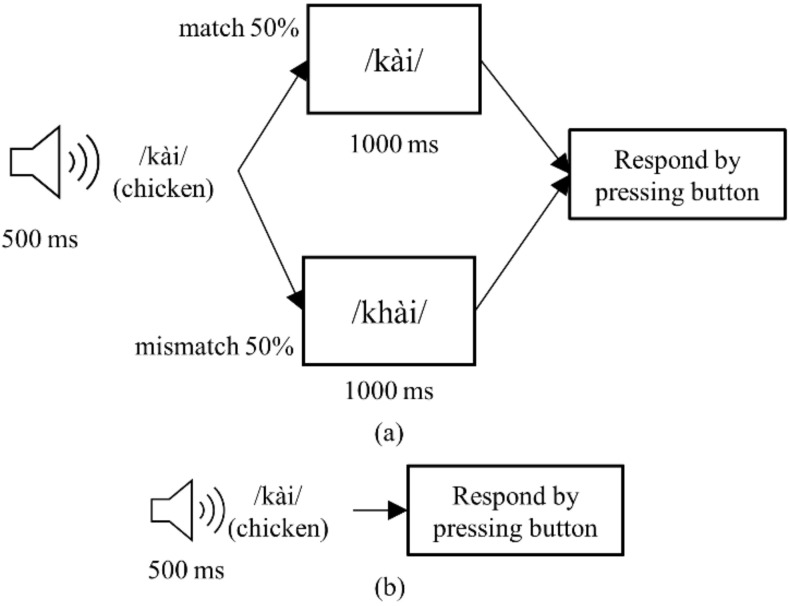
Event sequence for each trial in (**a**) Method 2; (**b**) Method 3.

Figure 2 shows the sequence of each trial in Methods 2 and 3. Method 2 was slightly adjusted from the method that was proposed by Morikawa et al. [26]. Each trial started by playing a word through the laptop speaker for 500 ms then showing a picture for 1000 ms. In 40 out of 80 trials, the picture was of the word that was played, spelled out using a standard font. In the remaining trials, the picture was of another word. The participants were required to press ‘1’ on the keyboard if they think the picture matched the word they heard and press ‘2’ otherwise.

Method 3 was an active oddball paradigm. In each trial, a word was played through the laptop speaker for 500 ms. On 80% of the trials (120 trials), the standard stimulus /kài/ was played. On the remaining 20% of the trials (30 trials), the deviant stimulus /k^h^ài/ was played. The participants were required to press ‘1’ on the keyboard when they heard the standard stimulus /kài/ and press ‘2’ when they heard the deviant stimulus /k^h^ài/.

### 2.3. EEG Recording and Analysis

**Figure 3:**
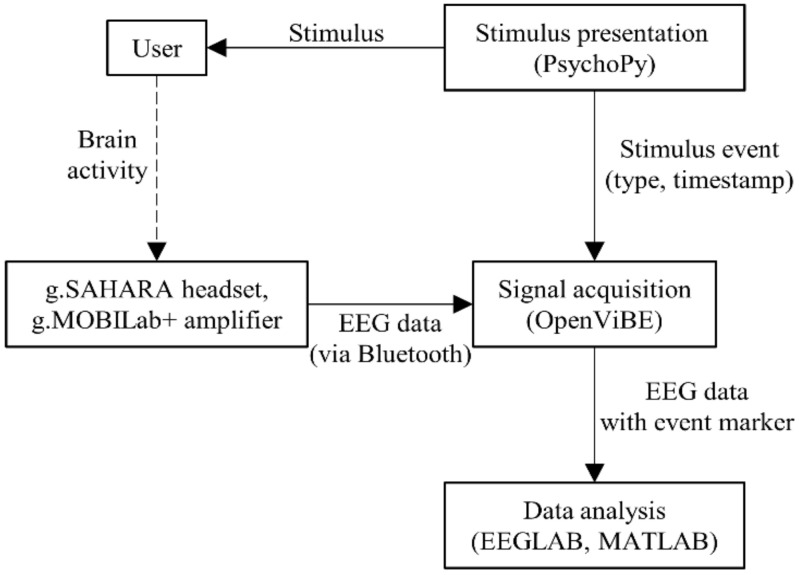
Overall EEG recording system [39].

Figure 3 shows the overall EEG recording system. EEG was recorded from eight positions (Fz, Cz, C3, C4, Pz, P3, P4, and Oz) according to the 10–20 system [4] using a g.SAHARA headset and a g.MOBIlab+ amplifier (g.tec medical engineering, Schiedlberg, Austria). The positions were chosen based on the scalp distribution of the ERP components that we expected to see. These included the MMN, P3, and LPP components. MMN had a frontocentral distribution and is prominent at Fz or Cz. P3 and LPP were reported to be most prominent at the parietal area. The signals were recorded with a sampling rate of 256 Hz.

The signal acquisition software, OpenViBE 3.0.0 [34], received the EEG signals via Bluetooth and combined the signals with the stimulus event data from the stimulus presentation software, PsychoPy 2020.1.2 [33], before outputting the EEG data with an event marker to be analyzed further. The EEG data was filtered with a bandpass filter. For stability and robustness, the filter used was an 847 point FIR filter with 1–40 Hz passband. It had transition frequencies of 1 Hz, −6 dB corner frequencies at 0.5 Hz and 40.5 Hz, and 50 dB attenuation at stopband. Then, it was passed through an EEGLAB [35] plugin function, clean_rawdata [36] to remove artifacts. The clean_rawdata function cleaned the EEG signals using the artifact subspace reconstruction (ASR) method [37]. The function rejected and reconstructed artifacts with variance more than 30 standard deviations (the cutoff parameter k = 30 was chosen according to [38]) away from the clean portions of the data. After that, the data were re-referenced to the common average reference and were extracted into epochs. For Method 1a, 1b, 1c, and 2, the interval from 0 to 900 ms after visual stimulus onset was extracted. For Method 3 the interval from 0 to 400 ms after stimulus onset was extracted. The −100 to 0 ms interval was used as a baseline. After removing the artifacts and extracting epochs, the data were manually inspected. Datasets that had less than half the original number of trials for each condition were excluded from further analysis.

Table 1 shows the amount of remaining data after preprocessing. For Method 1a, data from 9 subjects remained, including 328 epochs in the match condition and 320 epochs in the mismatch condition. For Method 1b, data from 10 subjects remained, including 389 epochs in the match condition and 1193 epochs in the mismatch condition. For Method 1c, data from 7 subjects remained, including 312 epochs in the match condition and 260 epochs in the mismatch condition. For Method 2, data from 25 subjects remained, including 881 epochs in the match condition and 879 epochs in the mismatch condition. For Method 3, data from 26 subjects remained, including 2795 epochs in the standard condition and 706 epochs in the deviant condition.

**Figure 4:**
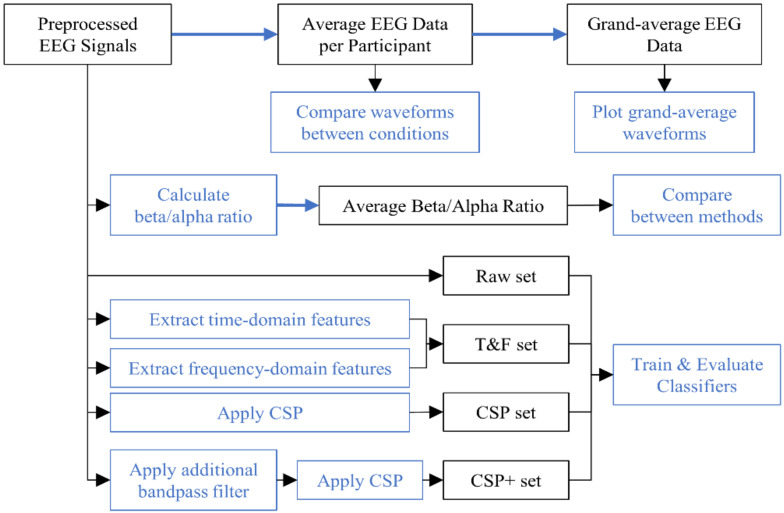
Overall EEG data processing. The black outlined boxes represent data while blue outlined boxes represent processes. Bold blue arrows represent averaging.

Figure 4 shows the overall EEG data processing. The EEG data were averaged according to match/mismatch or deviant/standard condition for each participant. The difference in waveforms between conditions were investigated by applying a paired *t*-test to the averaged EEG data for each participant in order to account for inter-subject variability. As beta and alpha waves were reported to be associated with attention [40], an average beta/alpha ratio was used to investigate the level of attention that was paid to each method. For each epoch, a beta/alpha ratio was calculated irrespective of the condition. Then, an average beta/alpha ratio was calculated for each participant and method. The Kruskal–Wallis test was applied to investigate the difference between beta/alpha ratios of each method. Post hoc analyses using Bonferroni correction for multiple comparisons were conducted when appropriate.

To classify between the conditions, several features were extracted from each epoch of EEG data. There were three types of features that were utilized including raw features, time-domain features, and frequency-domain features. The raw features were taken directly from the preprocessed data of each channel. The time-domain and frequency-domain features were extracted from two intervals in each trial. These intervals were 100–250 ms and 250–400 ms after stimulus onset for Method 3. For other methods, these intervals were 200–400 ms and 500–800 ms after visual stimulus onset. The intervals were chosen based on the result of the paired *t*-test and the expected ERP components for each method. The time-domain features included mean amplitude, variance, peak amplitude (maximum absolute amplitude), peak latency, maximal peak/amplitude ratio (MP ratio), positive area, and negative area. The frequency-domain features consisted of power in six frequency bands including Delta (1–4 Hz), Theta (4–8 Hz), Alpha (8–13 Hz), Beta (13–30 Hz), Gamma (30–40 Hz), and total band (1–40 Hz). A common spatial pattern (CSP) was applied to extract the CSP features. The extracted features were combined into four feature sets including raw features (Raw), time- and frequency-domain features (T&F), CSP features (CSP), and CSP features that were obtained after applying an additional bandpass filter (CSP+). The additional filter was a 16th order IIR filter with 1–30 Hz passband. It had 0.1 dB passband ripple and 60 dB attenuation at stopband. Each feature set was used to train and evaluate classifiers.

**Figure 5:**
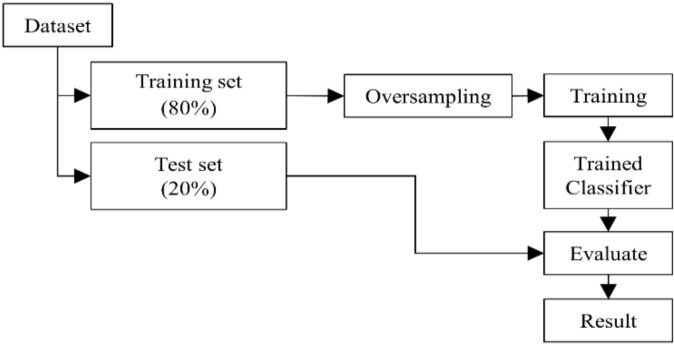
Process of training and evaluating the classifiers in each fold of the 5-fold cross-validation framework.

Figure 5 shows the process of training and evaluating classifiers in each fold of the 5-fold cross-validation framework. The dataset was separated into a training set (containing 80% of the data) and a test set (containing 20% of the data) with both sets having the same proportion of match/mismatch or standard/deviant conditions. Then, the features were extracted for both sets as described in the previous paragraph. For the CSP or CSP+ feature set, the training set was used to learn the CSP matrix, which was then applied to both the training set and test set. No data from the test set was used while learning the CSP matrix to prevent data leakage. After that, for the training set, the minority class (condition with lower number of epoch) was oversampled using random oversampling to achieve a 1:1 proportion between the conditions in the training data. Then, the training data were used to train either a linear discriminant analysis (LDA) or a support vector machine (SVM) classifier to classify whether the epoch was from a match or mismatch (or, for Method 3, standard or deviant) condition. The classifier was later evaluated using the test data and the results were recorded. The results from each fold were averaged and the classification accuracy was calculated from the number of correctly classified trials over the total number of trials.

The average classification accuracies across the participants for each method and feature set were calculated and compared. The comparison was done using the Kruskal–Wallis test. An analysis of variance (ANOVA) was used to compare the average accuracy between each feature set. Additionally, the classification accuracy that was obtained from the SVM and LDA classifiers trained using the same feature set were compared using a paired *t*-test. Post hoc analyses using Bonferroni correction for multiple comparisons were conducted when appropriate.

## 3. Results

### 3.1. Behavioral Measures

Table 2 shows the average behavioral accuracy and response time for each method. The participants answered correctly most of the time in every method (98.47%, 98.54%, 99.29%, 98.55%, and 98.77% of the cases for Method 1a, 1b, 1c, 2, and 3 respectively). The participants rarely failed to answer correctly (less than 2% of the cases in every method). The average response time for each method was 0.672 s, 0.645 s, 0.473 s, 0.518 s, and 0.697 s, respectively. A one-way ANOVA showed that there was a significant difference in the average response time between at least two groups (F(4,72) = 3.68, *p* = 0.0088). Post hoc analyses using Bonferroni correction indicated a significant difference in the average response time between Method 2 and 3 (*p* = 0.0202).

### 3.2. ERP Waveforms

**Figure 6:**
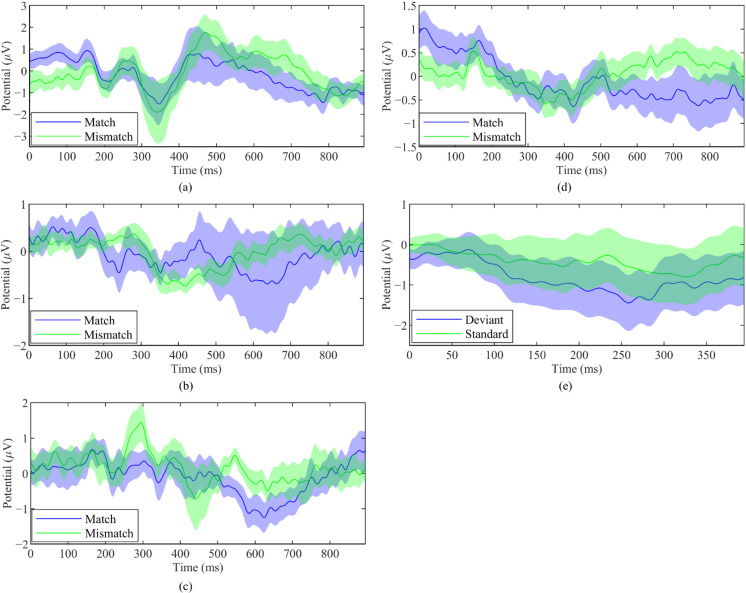
Grand-average waveforms with standard error at Pz (**a**) Method 1a; (**b**) Method 1b; (**c**) Method 1c; (**d**) Method 2; (**e**) Method 3.

Figure 6 shows the grand-average waveforms with standard error at Pz for each method. In Method 1a, a positive wave was observed at approximately 450–800 ms after stimulus onset in the mismatch condition. In Method 1b, no clear difference between the match and mismatch conditions were observed. We observed two intervals with difference between conditions in Method 1c. Positive waves were observed at approximately 200–400 ms and 500–750 ms after stimulus onset in the mismatch condition. In Method 2, a positive wave was elicited at approximately 500–900 ms after stimulus onset in the mismatch condition. In Method 3, a negative wave was observed at approximately 100–300 ms after stimulus onset in the deviant condition.

**Figure 7:**
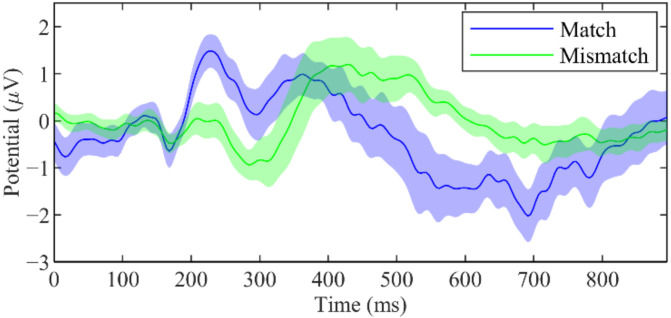
Grand-average waveforms with standard error at Fz in method 1b.

Despite having no clear difference between waveforms of match and mismatch condition at Pz, Method 1b had a more prominent difference at Fz. As illustrated in Figure 7, a positive wave was observed at approximately 200–400 ms after stimulus onset in the match condition. In the mismatch condition, a positive wave was elicited at approximately 400–800 ms after stimulus onset.

Table 3 shows the intervals with a significant difference, as indicated by a paired *t*-test, between the waveforms at Pz and Fz for each method. For Method 1a, a significant difference was found between the waveforms of the match and mismatch conditions during 23–78 ms, 141–145 ms, 160–168 ms, 488–512 ms, and 617–703 ms after stimulus onset at Pz. For Method 1b, a significant difference was found during 430–438 ms at Pz. However, at Fz, a significant difference was found during 195–281 ms, 309–336 ms, 492–609 ms, 617–625 ms, and 672–719 ms. For Method 1c, a significant difference was found during 535–555 ms at Pz and during 90–98 ms, 316–340 ms, 348–363 ms, 375–387 ms, 539–602 ms, 621–691 ms, and 699–711 ms at Fz. For Method 2, a significant difference was found during 4–39 ms, 59–90 ms, 191–203 ms, 633–762 ms, and 809–848 ms after stimulus onset at Pz. For Method 3, a significant difference was found between the waveforms of the standard and deviant conditions during 230–277 ms after stimulus onset at Pz.

The Kruskal–Wallis test indicated that no significant difference was found between the mean amplitude of the difference curve (mismatch condition–match condition) of Method 1a, 1b, and 1c during the 200–400 ms interval (H(2,23) = 1.48, *p* = 0.4778) and 500–800 ms interval (H(2,23) = 5.00, *p* = 0.0820) at Pz. However, at Fz, the Kruskal–Wallis test indicated that there was a significant difference in the mean amplitude of the difference curve between at least two methods during the 500–800 ms interval (H(2,23) = 13.23, *p* = 0.0013). Post hoc analyses using the Bonferroni correction revealed a significant difference between Method 1a and 1b (*p* = 0.0049) and between Method 1b and 1c (*p* = 0.0075). No significant difference was found during the 200–400 ms interval at Fz (H(2,23) = 3.14, *p* = 0.2082).

### 3.3. Classification

Table 4 compares the average classification accuracy that was achieved by the SVM and LDA classifiers. Both classifiers were trained using the same set of features (time- and frequency-domain features). The LDA performed better in Method 1a and 2. Although, a significant difference was found only for Method 2 (t(24) = −2.49, *p* = 0.0203). The SVM performed better in the remaining methods with significant difference for Method 1b (t(9) = 5.55, *p* = 0.0004) and 3 (t(25) = 5.01, *p* = 0.00004). No significant difference was found between the classifier type for Method 1a (t(8) = −1.79, *p* = 0.1112) and 1c (t(6) = 1.42, *p* = 0.2061).

**Figure 8:**
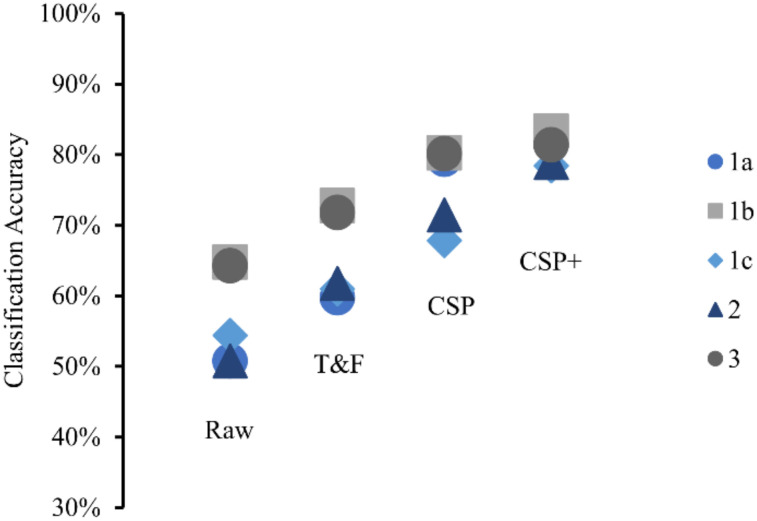
Comparison of the average accuracy between the different feature sets in each method.

Table 5 and Figure 8 shows the average accuracy from different feature sets for each method when using SVM. Overall, using raw features produced the worst average accuracy. Using the time- and frequency-domain features resulted in slightly better accuracy. Applying CSP to transform the raw features caused the average accuracy to rise further. The highest average accuracy for each method was obtained when using the CSP+ feature set. A one-way ANOVA indicated that there was a significant difference in accuracy between at least two feature sets (F(3,304) = 83.18, *p* = 2.5765 × 10^−39^). After post hoc comparison, significant difference was found between each pair of feature sets (*p* = 6.0125 × 10^−7^ between Raw and T&F sets, *p* = 3.4121 × 10^−25^ between Raw and CSP sets, *p* = 1.416 × 10^−35^ between Raw and CSP+ sets, *p* = 1.6078 × 10^−8^ between T&F and CSP sets, *p* = 1.7061 × 10^−16^ between T&F and CSP+ sets, and *p* = 0.028032 between CSP and CSP+ sets).

**Figure 9:**
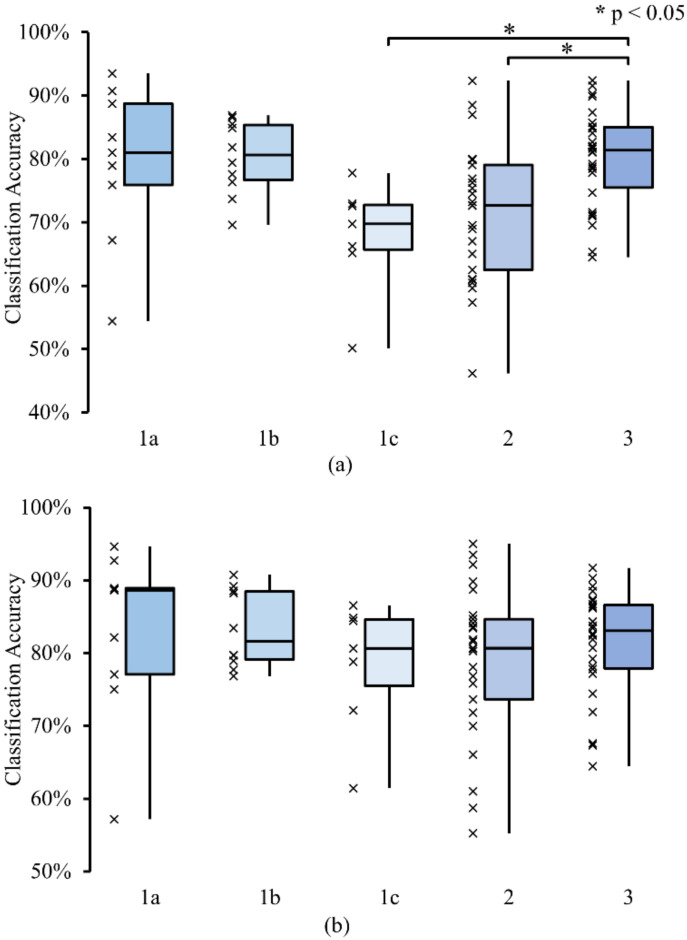
Box plot of the classification accuracy of each method using support vector machine (SVM) classifier (**a**) when using CSP feature set; (**b**) when using CSP+ feature set. The classification accuracies for individual participants are shown as x marks on the left of each box. An asterisk (*) denotes a significant difference in the classification accuracy between methods as indicated by the Kruskal–Wallis test with Bonferroni correction for multiple comparisons (*p* < 0.05).

The average accuracy was compared for each method, as shown in Figure 9a. When using the CSP set, Method 1b and 3 achieved the highest classification accuracy (80.24% and 80.15%, respectively). Method 1a performed slightly worse (79.31%), followed by Method 2 (71.48%). The lowest accuracy was obtained from Method 1c (67.80%). The Kruskal–Wallis test showed that there was a significant difference in the average accuracy between at least two methods (H(4,72) = 16.98, *p* = 0.002). Post hoc analyses using Bonferroni correction revealed a significant difference in average accuracy between Method 1c and 3 (*p* = 0.0378) and between method 2 and 3 (*p* = 0.0242). When using the CSP+ set, the average accuracy increased slightly; a similar trend was observed, although the differences were less pronounced. As shown in Figure 9b, the highest accuracy was obtained from Method 1b (83.31%), followed by Method 1a (82.79%) and Method 3 (81.35%). Method 2 and 1c produced slightly lower accuracy (78.90% and 78.41%, respectively). The result from the Kruskal–Wallis test indicated that no significant difference was found between the methods (H(4,72) = 2.63, *p* = 0.6218).

### 3.4. Attention

**Figure 10:**
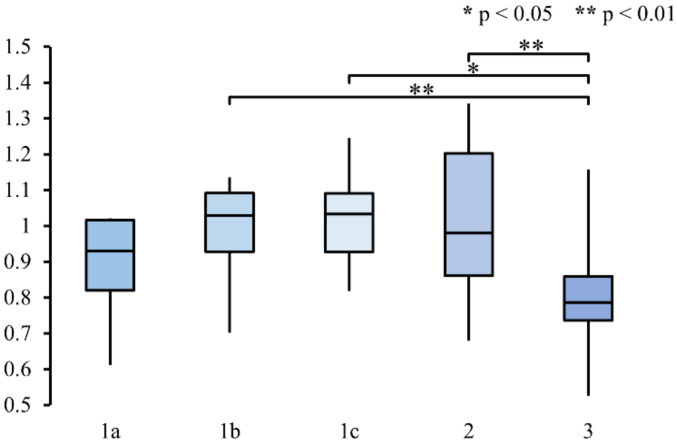
Box plot of the average beta/alpha ratio for each participant and method at Cz. Asterisks (*) denotes significant difference in beta/alpha ratio between the methods as indicated by the Kruskal–Wallis test with Bonferroni correction for multiple comparisons (* for *p* < 0.05 and ** for *p* < 0.01).

Figure 10 shows the average beta/alpha ratio at Cz for each method. Method 1b, 1c, and 2 have slightly higher beta/alpha ratio (0.9867, 1.0191, and 1.0092, respectively). Lower beta/alpha ratio was observed in Method 1a and 3 (0.8867 and 0.8002, respectively). The Kruskal–Wallis test revealed a significant difference between at least two methods (H(4,72) = 22.38, *p* = 0.0002). Post hoc analyses using Bonferroni correction showed a significant difference in average beta/alpha ratio between Method 1b and 3 (*p* = 0.0041), between Method 1c and 3 (*p* = 0.0366), and between Method 2 and 3 (*p* = 0.0012).

## 4. Discussion

Similar to Morikawa et al. [26], we found the LPP component during the mismatch condition in Method 1a, 1b, 1c, and 2. The component appeared as a positive waveform at approximately 500–800 ms after stimulus onset (Figure 6 and Figure 7). LPP (also called P600 [41] or P3b [28]) is elicited when the presented stimulus is different from the expectation [29]. We also found a P3 component during the match condition, as seen from the positive waveform between 300–400 ms after stimulus onset (Figure 6 and Figure 7). However, the difference in waveform was only significant in Method 1b and 1c. P3 is thought to be related to updating a mental representation of the incoming stimulus [28]. This component can be observed in various tasks such as oddball tasks [42,43], go/no-go or stop signal tasks [44,45,46], or identification tasks [47] during stimulus discrimination. P3 can be affected by stimulus probability and relevancy to the task. Method 1b included four pictures, from which the participants were required to select the correct one. This lower probability of the match stimulus resulted in a much clearer P3 component compared to all other methods. This result is in line with the findings that found increased P3 amplitude for rare stimuli compared to frequent stimuli [48,49]. We observed an MMN in response to deviant stimulus in Method 3 at approximately 100–300 ms after stimulus onset. This is consistent with the results from other works that utilizes MMN [13,15].

To classify between match/mismatch conditions, several feature sets were tested. The raw features produced the lowest classification accuracy compared to the other feature sets (Table 5). Combining the time-domain features and frequency-domain features into a feature set improved the accuracy slightly (Figure 8). However, classification accuracy from the time- and frequency-domain feature sets were below 80%. Thus, CSP was used to transform the raw features into a CSP feature set. An additional bandpass filter was applied to further improve the accuracy. CSP with the additional filter feature set produced the best accuracy compared to other feature sets. With this set, we obtained over 80% accuracy from Method 1a, 1b, and 3. This result is comparable to Morikawa et al. [26] which obtained over 80% accuracy when at least four trials were averaged.

Method 1b utilized four pictures to better differentiate between conditions. Thus, we expected this to result in much better classification accuracy compared to the other methods. When using the CSP+ feature set, we found that Method 1b performed best with 83.31% average classification accuracy. Method 1a and 3 also produced comparable average accuracies although with more variability of individual accuracies (Figure 9). Method 1c and 2 achieved slightly lower average accuracies (Figure 9). However, no significant difference between the average accuracy of each method was found when using this feature set. It is worth noting that this difference in accuracy between the methods was more pronounced when using Raw, T&F, or CSP feature sets (Figure 8). For example, the difference between the highest and lowest average accuracies (between Method 1b and 1c) increased from 4.9% with the CSP+ set to 12.44% with the CSP set. When using the CSP feature set, a significant difference was found between the average accuracy of Method 3 and 1c and between Method 3 and 2.

Despite being very similar to Method 1a, Method 1c produced the lowest accuracy compared to the rest. This went against our initial hypothesis that adding a prior section to set the expectation would increase the accuracy. This might be because of the participants getting confused as the two sections had different instructions. Method 2 was expected to achieve the same level of accuracy as experiment 1 in Morikawa et al. [26] as the design was similar. However, Method 2 achieved slightly lower accuracy at 78.90% compared to over 80% accuracy in Morikawa et al. [26]. This disparity might be because Morikawa et al. [26] used letters while our method used words. The participants had to evaluate the spelling of the words, which is relatively harder than identifying letters. This might cause more variation in ERP latency as each participant evaluates spelling using different techniques. Some people consider the spelling of the whole word while some people only look at the consonant. When averaged, this latency variation could cause ERP components to become less prominent.

To investigate the attention level in each method, we inspected the power in the beta and alpha bands. Higher attention is associated with an increase in beta power and a decrease in alpha power [40]. Thus, we used beta/alpha ratio to represent the attention level. Method 3, which included only auditory stimuli, had the lowest beta/alpha ratio (Figure 10). The beta/alpha ratio of this method also had lower variability compared to the other methods, as can be seen from the shorter distance between the first and third quartile in the box plot (Figure 10). This lower ratio indicates that less attention was paid to the task. A significant difference was found between Method 3 and Method 1b, 1c, and 2 (Figure 10). The latter three methods utilized visual stimuli which resulted in higher attention being paid to the task. This is in line with how higher activation was observed in a visual attention task compared to an auditory attention task [50]. Compared to the other methods, Method 3 required less attention from the participants.

Method 1a and 1b used pictures matching the meaning of the words. This is similar to the method that audiologists use to assess speech discrimination in children. This should make it easier to compare the results from the ERP methods and conventional methods. Furthermore, existing materials [19,20,51] can be conveniently adapted for the ERP methods. Between these two methods, Method 1a used less time per trial while having comparable accuracy to Method 1b. Furthermore, Method 1b required the participants to remember the presentation order of the correct picture, which might make this method more sensitive to errors in some population groups such as elderly patients with cognitive decline. On the other hand, Method 3 was easier to perform. It required less attention than Method 1a and 1b, as seen from the lower beta/alpha ratio (Figure 10). Also, with some adjustment, this method could be performed using passive listening. Passive listening does not require the participants to pay attention to what they are listening to. Participants can even engage in other light activity such as reading or watching a video while listening. This means that it can be used with a wider group of people, including very young children or uncooperating patients. However, this method might get more time consuming as more word pairs are added to the test. All things considered, we recommend using either Method 1a or 3 depending on the target population. Method 1a might be a better choice in cases where existing materials can be adapted easily. However, Method 3 might be better for participants who have difficulties following instructions.

The participants answered correctly in most of the cases in all methods, in line with the fact that all the participants had normal hearing. The response time varied between methods with the highest average time in Method 3. This was surprising as Method 3 was the only method that utilized auditory stimulus only. Auditory stimulus usually had lower response time than visual stimulus [52,53]. It was possible that, because the words sounded very similar, they were difficult to differentiate, while the picture of word meaning did not resemble the other, making it easier to discern the correct answer in the visual methods. Thus, the average response time was higher in the auditory method and lower in the visual methods. The participants’ opinion on each method varied. Some participants preferred the auditory-only method as they did not have to focus their attention. Others preferred having visual stimuli because they could differentiate between the conditions more easily with visual stimuli.

Using a cap with eight electrodes, our setup time was approximately 10–20 min. During this time, the participants were given instructions for each method. Some participants requested a very detailed explanation which could extend this setup time further. The two most promising methods, Method 1a and 3, took approximately five minutes to complete for one contrast repeated 40 times. During offline analysis, the result for these methods was obtained in under 10 s for each participant. In a real situation, more word pairs should be used but with lower repetition for each pair. Hypothetically, for 50 pairs, Method 1a would take under 10 min to complete if each pair was used twice. Method 3, using 20 trials per pair, could be completed in approximately 30 min. The experiment time for Method 3 could be reduced by adjusting the design. For example, using a double or multi-feature oddball paradigm [15,22]. The analysis time would vary depending on the hardware performance but should not take more than 30 min per patient. Overall, a speech discrimination assessment system using these methods could be done in under one hour.

The ERP-based methods of speech discrimination assessment that were investigated in this work could potentially be developed into an automated system that acts as a pre-screening tool. EEG is gradually becoming more accessible and user-friendly, as can be seen from the increased availability of consumer EEG devices such as Emotiv Insight (Emotiv, San Francisco, CA, USA), NeuroSky Mindwave (NeuroSky, San Jose, CA, USA), OpenBCI headset (OpenBCI, Brooklyn, NY, USA), etc. (for review of low-cost EEG headsets, see [54]). Such consumer EEG devices can be used by trained healthcare professionals (e.g., nurses, practical nurses, or health technicians) in local health centers to pre-screen patients using the automatic system. Then, if the result indicates speech discrimination problems, an in-person or telemedicine appointment with an audiologist may be scheduled. Employing such a system would make the assessment more accessible, especially in areas with a shortage of audiologists.

## 5. Limitations

This study has some limitations that warrant future research. Although the sample size used is not dissimilar to those in various BCI studies [55,56,57], it can still be considered rather small, especially for Method 1a, 1b, and 1c which involved 10 participants. Further studies with a larger sample size may help confirm the accuracy of the proposed methods. Furthermore, this experiment used only one pair of words with a consonant contrast to test the different methods. To cover all the meaningful contrast types in a language, a comprehensive word list [19,20,21,58] could be included. For example, in Thai, which is a tonal language, the word list should include all vowel and consonant groups with varied tones [19]. The method could also be further validated with evaluations that are made by audiologists in patients with hearing loss and other associated diseases. In addition, all the methods that were included in this work used active listening. The feasibility of a method utilizing passive listening should also be investigated. Näätänen et al. [11] suggested passive listening might produce clearer MMN waveform. However, there has not yet been any confirmation of any effect on classification accuracy. Passive listening allows the assessment to be applied to a much wider group of people. Thus, a passive listening method could be very useful if a comparable level of classification accuracy could be achieved.

## 6. Conclusions

This research compared several ERP-based methods for assessing speech discrimination. Of these, two methods are recommended. Both achieved 80% classification accuracy and required less time or effort than other methods. The first method used picture representing word meaning which allowed for easy adaptation of existing materials to be incorporated into the assessment. P3 and LPP were observed and used to classify whether the sound and picture matched. This method achieved 82.79% accuracy. The second recommended method used auditory stimuli only. MMN was elicited in response to the deviant stimuli and was used to classify between standard and deviant stimulus, achieving 81.35% accuracy. This method took longer to complete than the first but required less attention from participants. It is well-suited for use in cases where pictures are not available or where participants have difficulties following instructions.

## Figures and Tables

**Table 1:** Remaining data after preprocessing.

Method	Remaining Participants	Remaining Epochs
1a	9	Match: 328 Mismatch: 320
1b	10	Match: 389 Mismatch: 1193
1c	7	Match: 312 Mismatch: 260
2	25	Match: 881 Mismatch: 879
3	26	Standard: 2795 Deviant: 706

**Table 2:** Mean (standard error) behavioral accuracy and response time for each method.

Method	Average Correct Answers (%)	Average Response Time (s)
1a	98.47 (0.62)	0.672 (0.12)
1b	98.54 (0.62)	0.645 (0.05)
1c	99.29 (0.37)	0.473 (0.03)
2	98.55 (0.44)	0.518 (0.03)
3	98.77 (0.48)	0.697 (0.04)

**Table 3:** Intervals with a significant difference at Pz and Fz for each method.

Method	Interval with Significant Difference (at Pz)	Interval with Significant Difference (at Fz)
1a	23–78 ms	
	141–145 ms	
	160–168 ms	
	488–512 ms	
	617–703 ms	
1b	430–438 ms	
		195–281 ms
		309–336 ms
		492–609 ms
		617–625 ms
		672–719 ms
1c	535–555 ms	
		90–98 ms
		316–340 ms
		348–363 ms
		375–387 ms
		539–602 ms
		621–691 ms
		699–711 ms
2	4–39 ms	
	59–90 ms	
	191–203 ms	
	633–762 ms	
	809–848 ms	
3	230–277 ms	

**Table 4:** The mean (standard error) classification accuracy from support vector machine (SVM) and linear discriminant analysis (LDA) classifiers for each method when using the time- and frequency-domain feature set.

Method	Classification Accuracy	
	SVM	LDA
1a	59.71 (4.85)	63.07 (4.03)
1b	72.73 (2.21)	62.01 (1.98)
1c	60.99 (2.78)	57.56 (3.16)
2	61.75 (2.03)	64.23 (2.11)
3	71.78 (1.94)	65.74 (1.95)

**Table 5:** The mean (standard error) classification accuracy from each feature set for each method when using SVM classifier.

Method	Classification Accuracy (%)			
	Raw	T&F	CSP	CSP+
1a	50.77 (2.23)	59.71 (4.85)	79.31 (4.11)	82.79 (3.90)
1b	64.77 (2.49)	72.73 (2.21)	80.24 (1.87)	83.31 (1.70)
1c	54.39 (2.60)	60.99 (2.78)	67.80 (3.36)	78.41 (3.37)
2	50.74 (1.54)	61.75 (2.03)	71.48 (2.16)	78.90 (2.10)
3	64.27 (0.99)	71.78 (1.94)	80.15 (1.52)	81.35 (1.43)

## Data Availability

The data presented in this study are openly available in FigShare at https://doi.org/10.6084/m9.figshare.16786618.v2 (accessed on 24 January 2022).
